# Bis[6-(3,5-dimethyl-1*H*-pyrazol-1-yl-κ*N* ^2^)picolinato-κ^2^ *N*,*O*]manganese(II) bis­(3,5-dinitro­benzoic acid) solvate

**DOI:** 10.1107/S1600536808016759

**Published:** 2008-06-07

**Authors:** Fei-Long Hu, Xian-Hong Yin, Wei-Qiang Luo, Kai Zhao, Cui-Wu Lin

**Affiliations:** a College of Chemistry and Ecological Engineering, Guangxi University for Nationalities, Nanning 530006, People’s Republic of China

## Abstract

In the title complex, [Mn(C_11_H_10_N_3_O_2_)_2_]·2C_7_H_4_N_2_O_6_, the Mn^II^ atom has a disorted octa­hedral coordination formed by four N and two O atoms of two *mer*-6-(3,5-dimethyl-1*H*-pyrazol-1-yl)picolinate ligands (DMPP). Each of the two symmetry-independent 3,5-dinitro­benzoic acid mol­ecules is linked to the mol­ecule of the complex *via* a hydrogen bond involving its carboxylic H atom and one of the DMPP ligands of the complex. However, in one of the DMPP ligands, the non-coordinated carbonyl O atom serves as the hydrogen-bond acceptor, whereas in the second ligand it is the Mn-coordinated O atom which is involved in the hydrogen bonding.

## Related literature

For related literature, see: Feng *et al.* (2008 ▶); Yin *et al.* (2007 ▶); Zhao *et al.* (2007 ▶).
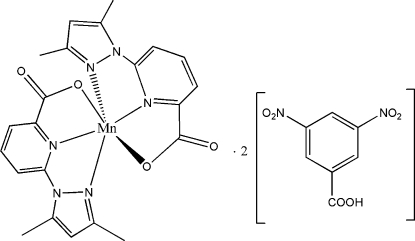


## Experimental

### 

#### Crystal data

[Mn(C_11_H_10_N_3_O_2_)_2_]·2C_7_H_4_N_2_O_6_
*M* *_r_* = 911.62
Triclinic, 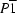

*a* = 10.4291 (10) Å
*b* = 13.7472 (18) Å
*c* = 15.736 (2) Å
α = 69.180 (1)°
β = 88.085 (2)°
γ = 67.968 (1)°
*V* = 1941.7 (4) Å^3^
*Z* = 2
Mo *K*α radiation
μ = 0.43 mm^−1^
*T* = 298 (2) K
0.55 × 0.50 × 0.38 mm

#### Data collection

Bruker SMART CCD area-detector diffractometer
Absorption correction: multi-scan (*SADABS*; Sheldrick, 1996 ▶) *T*_min_ = 0.798, *T*_max_ = 0.854
10090 measured reflections
6713 independent reflections
4919 reflections with *I* > 2σ(*I*)
*R*_int_ = 0.029

#### Refinement

*R*[*F* ^2^ > 2σ(*F* ^2^)] = 0.045
*wR*(*F* ^2^) = 0.127
*S* = 1.03
6713 reflections
570 parameters
H-atom parameters constrained
Δρ_max_ = 0.31 e Å^−3^
Δρ_min_ = −0.45 e Å^−3^

### 

Data collection: *SMART* (Siemens, 1996 ▶); cell refinement: *SAINT* (Siemens, 1996 ▶); data reduction: *SAINT*; program(s) used to solve structure: *SHELXS97* (Sheldrick, 2008 ▶); program(s) used to refine structure: *SHELXL97* (Sheldrick, 2008 ▶); molecular graphics: *SHELXTL* (Sheldrick, 2008 ▶); software used to prepare material for publication: *SHELXTL*.

## Supplementary Material

Crystal structure: contains datablocks I, global. DOI: 10.1107/S1600536808016759/ya2067sup1.cif

Structure factors: contains datablocks I. DOI: 10.1107/S1600536808016759/ya2067Isup2.hkl

Additional supplementary materials:  crystallographic information; 3D view; checkCIF report

## Figures and Tables

**Table:** 

Mn1—O1	2.1837 (19)
Mn1—O3	2.167 (2)
Mn1—N1	2.207 (2)
Mn1—N3	2.215 (2)
Mn1—N4	2.222 (2)
Mn1—N6	2.282 (2)

**Table:** 

O3—Mn1—O1	93.24 (8)
O3—Mn1—N1	106.48 (7)
O1—Mn1—N1	72.91 (7)
O3—Mn1—N3	96.33 (8)
O1—Mn1—N3	143.17 (7)
N1—Mn1—N3	70.27 (8)
O3—Mn1—N4	72.54 (7)
O1—Mn1—N4	96.62 (7)
N1—Mn1—N4	169.48 (8)
N3—Mn1—N4	120.18 (8)
O3—Mn1—N6	141.46 (7)
O1—Mn1—N6	97.83 (8)
N1—Mn1—N6	112.06 (8)
N3—Mn1—N6	96.48 (8)
N4—Mn1—N6	69.58 (8)

**Table 2:** Hydrogen-bond geometry (Å, °)

*D*—H⋯*A*	*D*—H	H⋯*A*	*D*⋯*A*	*D*—H⋯*A*
O6—H6⋯O4	0.82	1.76	2.575 (3)	171
O12—H12⋯O1	0.82	1.76	2.552 (3)	162

**Fig. 1.**
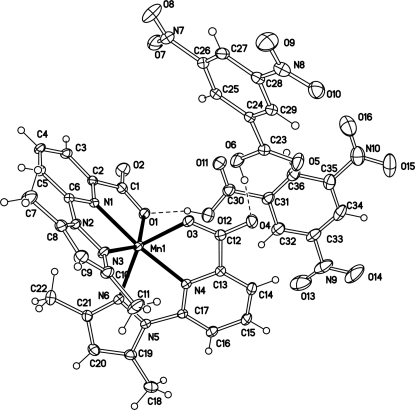
The structure of the title compound (I) showing 50% probability displacement ellipsoids and the atom-numbering scheme; H atoms are represented as small circles of arbitrary radius. The bonds in the coordination sphere of the Mn1 atom are drawn as solid sticks; the rest of the bonds in the ligands and dinitrobenzoic moelecules are depicted as hollow sticks; H-bonds are shown as dashed lines.

## supplementary crystallographic information

### Comment

Recently we reported the crystal structures of bis(6-(3,5-dimethyl-1*H*-pyrazol-1-yl)picolinato)zinc(II) trihydrate (Yin *et al.*, 2007), bis[3-chloro-6-(3,5-dimethyl-1*H*-pyrazol-1-yl)picolinato]cobalt(II) 2.5-hydrate (Zhao *et al.*, 2007), and bis(6-(3,5-dimethyl-1*H*-pyrazol-1-yl)picolinato)manganese(II) trihydrate (Feng *et al.*, 2008), In continuation of these studies, we report here the crystal structure of bis(3,5-dinitrobenzoic acid) solvate of bis(6-(3,5-dimethyl-1*H*-pyrazol-1-yl)picolinato)manganese(II).

The Mn1 atom (Fig. 1) has a distorted octahedral environment formed by four N and two O atoms of two *mer*-coordinated 6-(3,5-dimethyl-1*H*-pyrazol-1-yl)picolinato (DMPP) ligands. The Mn—O and Mn—N bond lengths (Table 1) are close to those found in similar compounds, *e.g.* in the most recently studied trihydrate manganese complex reported by Feng *et al.* (2008).

The crystal structure is built of the isolated units, each of them made up of the complex molecule and two 3,5-dinitrobenzoic acid molecules. Each of the 3,5-dinitrobenzoic acid molecules is linked to the molecule of the complex *via* H-bond involving its carboxylic H atom and one of the DMPP ligands of the complex, however in one of the DMPP ligands the non-coordinated carbonyl oxygen O4 serves as the H-bond acceptor, whereas in the second ligand it is the Mn-coordinated O1 atom, which is involved in the H-bonding (Table 2).

### Experimental

6-(3-Chloro-(3,5-dimethyl-1*H*-pyrazol-1-yl))picolinic acid, 3,5-dinitrobenzoic acid and MnCl_2_.6H_2_O are available commercially and were used without further purification. 1 mmol (250 mg) of 6-(3-chloro-(3,5-dimethyl-1*H*-pyrazol-1-yl))picolinic acid and 1 mmol (212 mg) of 3,5-dinitrobenzoic acid were dissolved in 15 ml of anhydrous ethyl alcohol (15 ml). The mixture was stirred to give a clear solution, then 0.5 mmol (142 mg) of MnCl_2_.6H_2_O in 10 ml of anhydrous alcohol were added. The suspension was stirred for *ca* 4 hrs and filtered. After keeping the filtrate in air for one week, colorless prisms of the title compound precipitated. The crystals were isolated, washed with alcohol three times and dried in a vacuum desiccator using silica gel (yield 75%). Elemental analysis: found C, 47.23; H, 3.30; N, 15.13.; calc. for C_36_H_28_MnN_10_O_16_: C, 47.43; H, 3.10; N, 15.36.

### Refinement

H atoms bound to the C atoms were positoned geometrically and refined using a riding model with C—H = 0.93–0.96 Å and U_iso_(H) = 1.5U_eq_(C) for methyl H atoms and U_iso_(H) = 1.2U_eq_(C) for H atoms in aromatic rings. Two H atoms bound to the O atoms were located in the difference Fourier map and then refined using riding model, the idealized O—H distance of 0.82 Å, and  U_iso_(H) = 1.5U_eq_(O).

### Crystal data

**Table:**

[Mn(C_11_H_10_N_3_O_2_)_2_]·2C_7_H_4_N_2_O_6_	*Z* = 2
*M**_r_* = 911.62	*F*_000_ = 934
Triclinic, *P*1	*D*_x_ = 1.559 Mg m^−^^3^
*a* = 10.4291 (10) Å	Mo *K*α radiation λ = 0.71073 Å
*b* = 13.7472 (18) Å	Cell parameters from 4193 reflections
*c* = 15.736 (2) Å	θ = 2.3–27.2º
α = 69.180 (1)º	µ = 0.43 mm^−^^1^
β = 88.085 (2)º	*T* = 298 (2) K
γ = 67.968 (1)º	Block, colourless
*V* = 1941.7 (4) Å^3^	0.55 × 0.50 × 0.38 mm

### Data collection

**Table:**

Bruker SMART CCD area-detector diffractometer	6713 independent reflections
Radiation source: fine-focus sealed tube	4919 reflections with *I* > 2σ(*I*)
Monochromator: graphite	*R*_int_ = 0.029
*T* = 298(2) K	θ_max_ = 25.0º
φ and ω scans	θ_min_ = 1.4º
Absorption correction: multi-scan(SADABS; Sheldrick, 1996)	*h* = −12→12
*T*_min_ = 0.798, *T*_max_ = 0.854	*k* = −16→16
10090 measured reflections	*l* = −18→17

### Refinement

**Table:**

Refinement on *F*^2^	Secondary atom site location: difference Fourier map
Least-squares matrix: full	Hydrogen site location: inferred from neighbouring sites
*R*[*F*^2^ > 2σ(*F*^2^)] = 0.045	H-atom parameters constrained
*wR*(*F*^2^) = 0.127	*w* = 1/[σ^2^(*F*_o_^2^) + (0.0574*P*)^2^ + 0.4835*P*] where *P* = (*F*_o_^2^ + 2*F*_c_^2^)/3
*S* = 1.03	(Δ/σ)_max_ = 0.001
6713 reflections	Δρ_max_ = 0.31 e Å^−^^3^
570 parameters	Δρ_min_ = −0.45 e Å^−^^3^
Primary atom site location: structure-invariant direct methods	Extinction correction: none

### Special details

**Table:**

Geometry. All e.s.d.'s (except the e.s.d. in the dihedral angle between two l.s. planes) are estimated using the full covariance matrix. The cell e.s.d.'s are taken into account individually in the estimation of e.s.d.'s in distances, angles and torsion angles; correlations between e.s.d.'s in cell parameters are only used when they are defined by crystal symmetry. An approximate (isotropic) treatment of cell e.s.d.'s is used for estimating e.s.d.'s involving l.s. planes.
Refinement. Refinement of *F*^2^ against ALL reflections. The weighted *R*-factor *wR* and goodness of fit *S* are based on *F*^2^, conventional *R*-factors *R* are based on *F*, with *F* set to zero for negative *F*^2^. The threshold expression of *F*^2^ > σ(*F*^2^) is used only for calculating *R*-factors(gt) *etc*. and is not relevant to the choice of reflections for refinement. *R*-factors based on *F*^2^ are statistically about twice as large as those based on *F*, and *R*- factors based on ALL data will be even larger.

### Fractional atomic coordinates and isotropic or equivalent isotropic displacement parameters (Å^2^)

**Table:**

	*x*	*y*	*z*	*U*_iso_*/*U*_eq_	
Mn1	0.63361 (4)	0.53951 (3)	0.25213 (2)	0.03494 (14)	
N1	0.6091 (2)	0.43850 (17)	0.39159 (13)	0.0313 (5)	
N2	0.8413 (2)	0.38572 (18)	0.42839 (13)	0.0357 (5)	
N3	0.8389 (2)	0.44519 (19)	0.33637 (14)	0.0394 (6)	
N4	0.6159 (2)	0.65388 (18)	0.10779 (13)	0.0326 (5)	
N5	0.6647 (2)	0.50244 (18)	0.06512 (13)	0.0363 (5)	
N6	0.6784 (2)	0.44054 (19)	0.15744 (14)	0.0399 (6)	
N7	0.6338 (3)	0.7621 (2)	0.67814 (18)	0.0599 (8)	
N8	0.8598 (3)	1.0309 (2)	0.5605 (2)	0.0562 (7)	
N9	0.1281 (4)	1.0940 (5)	−0.1595 (2)	0.0954 (14)	
N10	0.0870 (4)	1.2586 (3)	0.0755 (3)	0.0849 (10)	
O1	0.41227 (19)	0.57206 (16)	0.25620 (12)	0.0420 (5)	
O2	0.2485 (2)	0.5314 (2)	0.33903 (15)	0.0613 (6)	
O3	0.6057 (2)	0.70344 (16)	0.25314 (12)	0.0451 (5)	
O4	0.5467 (3)	0.88701 (18)	0.17175 (13)	0.0585 (6)	
O5	0.6033 (3)	1.0434 (2)	0.28162 (15)	0.0767 (8)	
O6	0.5769 (3)	0.8812 (2)	0.33545 (14)	0.0712 (7)	
H6	0.5687	0.8896	0.2814	0.107*	
O7	0.5504 (4)	0.7274 (2)	0.66235 (17)	0.0862 (9)	
O8	0.6893 (3)	0.7358 (3)	0.75365 (17)	0.0982 (10)	
O9	0.9080 (3)	1.0042 (3)	0.63827 (18)	0.0874 (9)	
O10	0.8765 (3)	1.1041 (2)	0.49409 (18)	0.0671 (6)	
O11	0.2409 (3)	0.8355 (2)	0.24857 (16)	0.0873 (9)	
O12	0.2601 (3)	0.7649 (2)	0.13889 (15)	0.0682 (7)	
H12	0.2933	0.7038	0.1815	0.102*	
O13	0.1350 (5)	1.0131 (4)	−0.1773 (2)	0.1319 (16)	
O14	0.1116 (4)	1.1865 (4)	−0.2162 (2)	0.1370 (15)	
O15	0.0689 (4)	1.3489 (3)	0.0138 (3)	0.1250 (13)	
O16	0.0784 (4)	1.2459 (3)	0.1555 (3)	0.1114 (12)	
C1	0.3686 (3)	0.5203 (2)	0.32928 (19)	0.0399 (7)	
C2	0.4808 (3)	0.4421 (2)	0.40902 (17)	0.0333 (6)	
C3	0.4560 (3)	0.3818 (2)	0.49408 (18)	0.0397 (7)	
H3	0.3666	0.3857	0.5059	0.048*	
C4	0.5677 (3)	0.3153 (2)	0.56142 (18)	0.0426 (7)	
H4	0.5538	0.2726	0.6191	0.051*	
C5	0.6996 (3)	0.3115 (2)	0.54414 (17)	0.0400 (7)	
H5	0.7752	0.2671	0.5891	0.048*	
C6	0.7153 (3)	0.3762 (2)	0.45728 (16)	0.0326 (6)	
C7	1.0062 (3)	0.2924 (3)	0.57596 (19)	0.0605 (9)	
H7A	0.9384	0.3311	0.6080	0.091*	
H7B	1.0962	0.2884	0.5928	0.091*	
H7C	1.0090	0.2174	0.5918	0.091*	
C8	0.9671 (3)	0.3553 (2)	0.47501 (18)	0.0427 (7)	
C9	1.0458 (3)	0.3950 (3)	0.4115 (2)	0.0504 (8)	
H9	1.1369	0.3871	0.4223	0.060*	
C10	0.9632 (3)	0.4497 (3)	0.32706 (19)	0.0455 (7)	
C11	0.9977 (4)	0.5078 (3)	0.2347 (2)	0.0704 (11)	
H11A	1.0396	0.4539	0.2061	0.106*	
H11B	1.0616	0.5406	0.2411	0.106*	
H11C	0.9141	0.5661	0.1975	0.106*	
C12	0.5758 (3)	0.7874 (2)	0.17932 (18)	0.0397 (7)	
C13	0.5764 (3)	0.7638 (2)	0.09248 (17)	0.0362 (6)	
C14	0.5378 (3)	0.8447 (2)	0.00519 (18)	0.0464 (7)	
H14	0.5123	0.9208	−0.0049	0.056*	
C15	0.5381 (3)	0.8095 (3)	−0.06682 (19)	0.0521 (8)	
H15	0.5115	0.8625	−0.1263	0.063*	
C16	0.5775 (3)	0.6968 (3)	−0.05140 (17)	0.0475 (8)	
H16	0.5763	0.6724	−0.0993	0.057*	
C17	0.6189 (3)	0.6209 (2)	0.03810 (16)	0.0337 (6)	
C18	0.7141 (4)	0.4711 (3)	−0.0843 (2)	0.0692 (11)	
H18A	0.6216	0.5099	−0.1160	0.104*	
H18B	0.7632	0.4067	−0.1004	0.104*	
H18C	0.7625	0.5213	−0.1012	0.104*	
C19	0.7066 (3)	0.4332 (3)	0.01624 (19)	0.0437 (7)	
C20	0.7459 (3)	0.3264 (3)	0.0790 (2)	0.0518 (8)	
H20	0.7787	0.2604	0.0668	0.062*	
C21	0.7286 (3)	0.3334 (2)	0.1647 (2)	0.0452 (7)	
C22	0.7617 (4)	0.2397 (3)	0.2570 (2)	0.0690 (10)	
H22A	0.8604	0.2066	0.2746	0.104*	
H22B	0.7311	0.1832	0.2540	0.104*	
H22C	0.7149	0.2696	0.3012	0.104*	
C23	0.6100 (3)	0.9592 (3)	0.34256 (19)	0.0449 (7)	
C24	0.6568 (3)	0.9360 (2)	0.43964 (17)	0.0386 (7)	
C25	0.6248 (3)	0.8595 (2)	0.51314 (18)	0.0417 (7)	
H25	0.5739	0.8207	0.5035	0.050*	
C26	0.6697 (3)	0.8422 (2)	0.60026 (18)	0.0448 (7)	
C27	0.7473 (3)	0.8953 (3)	0.61884 (19)	0.0480 (8)	
H27	0.7786	0.8807	0.6785	0.058*	
C28	0.7766 (3)	0.9718 (2)	0.54407 (19)	0.0436 (7)	
C29	0.7330 (3)	0.9929 (2)	0.45572 (18)	0.0429 (7)	
H29	0.7542	1.0449	0.4070	0.052*	
C30	0.2307 (3)	0.8463 (3)	0.1699 (2)	0.0560 (9)	
C31	0.1851 (3)	0.9618 (3)	0.0943 (2)	0.0524 (8)	
C32	0.1775 (3)	0.9741 (3)	0.0033 (2)	0.0584 (9)	
H32	0.1983	0.9115	−0.0130	0.070*	
C33	0.1384 (3)	1.0810 (4)	−0.0627 (2)	0.0646 (10)	
C34	0.1096 (3)	1.1753 (3)	−0.0417 (2)	0.0680 (11)	
H34	0.0862	1.2466	−0.0871	0.082*	
C35	0.1167 (3)	1.1597 (3)	0.0496 (2)	0.0611 (9)	
C36	0.1546 (3)	1.0540 (3)	0.1184 (2)	0.0569 (9)	
H36	0.1591	1.0457	0.1796	0.068*	

### Atomic displacement parameters (Å^2^)

**Table:**

	*U*^11^	*U*^22^	*U*^33^	*U*^12^	*U*^13^	*U*^23^
Mn1	0.0381 (2)	0.0356 (2)	0.0248 (2)	−0.01310 (19)	0.00740 (16)	−0.00572 (17)
N1	0.0349 (12)	0.0287 (12)	0.0300 (11)	−0.0121 (10)	0.0109 (9)	−0.0113 (9)
N2	0.0338 (12)	0.0396 (13)	0.0252 (11)	−0.0117 (10)	0.0073 (9)	−0.0056 (9)
N3	0.0372 (13)	0.0442 (14)	0.0286 (11)	−0.0159 (11)	0.0081 (9)	−0.0046 (10)
N4	0.0358 (12)	0.0327 (12)	0.0267 (11)	−0.0121 (10)	0.0058 (9)	−0.0096 (9)
N5	0.0424 (13)	0.0362 (13)	0.0272 (11)	−0.0131 (11)	0.0092 (9)	−0.0110 (10)
N6	0.0462 (14)	0.0368 (13)	0.0301 (11)	−0.0137 (11)	0.0112 (10)	−0.0080 (10)
N7	0.083 (2)	0.0513 (17)	0.0403 (16)	−0.0244 (16)	0.0222 (15)	−0.0146 (13)
N8	0.0551 (17)	0.0574 (18)	0.0615 (18)	−0.0171 (15)	0.0109 (14)	−0.0335 (15)
N9	0.071 (2)	0.134 (4)	0.042 (2)	−0.025 (3)	0.0057 (16)	−0.003 (2)
N10	0.068 (2)	0.065 (2)	0.104 (3)	−0.0218 (19)	−0.010 (2)	−0.015 (2)
O1	0.0384 (11)	0.0420 (11)	0.0366 (10)	−0.0129 (9)	0.0059 (8)	−0.0076 (9)
O2	0.0373 (12)	0.0760 (16)	0.0668 (15)	−0.0272 (12)	0.0097 (10)	−0.0169 (12)
O3	0.0650 (13)	0.0411 (11)	0.0289 (10)	−0.0225 (10)	0.0087 (9)	−0.0110 (9)
O4	0.0921 (18)	0.0396 (13)	0.0443 (12)	−0.0249 (12)	0.0048 (11)	−0.0164 (10)
O5	0.137 (2)	0.0535 (15)	0.0394 (12)	−0.0481 (16)	−0.0085 (13)	−0.0030 (11)
O6	0.134 (2)	0.0642 (16)	0.0371 (12)	−0.0586 (16)	0.0135 (14)	−0.0216 (11)
O7	0.143 (3)	0.0771 (19)	0.0665 (17)	−0.071 (2)	0.0429 (17)	−0.0312 (14)
O8	0.126 (3)	0.114 (2)	0.0384 (15)	−0.056 (2)	0.0121 (15)	−0.0002 (14)
O9	0.105 (2)	0.114 (2)	0.0646 (17)	−0.0570 (19)	0.0005 (15)	−0.0414 (16)
O10	0.0728 (17)	0.0604 (16)	0.0782 (17)	−0.0328 (14)	0.0170 (13)	−0.0304 (14)
O11	0.117 (2)	0.0644 (17)	0.0390 (14)	−0.0049 (16)	0.0073 (13)	−0.0050 (12)
O12	0.0737 (17)	0.0556 (15)	0.0514 (13)	−0.0116 (14)	−0.0046 (12)	−0.0063 (12)
O13	0.149 (4)	0.175 (4)	0.058 (2)	−0.050 (3)	0.0134 (19)	−0.042 (2)
O14	0.143 (3)	0.152 (3)	0.0457 (17)	−0.040 (3)	0.0029 (18)	0.024 (2)
O15	0.127 (3)	0.060 (2)	0.153 (3)	−0.035 (2)	0.000 (2)	−0.001 (2)
O16	0.128 (3)	0.082 (2)	0.111 (3)	−0.023 (2)	−0.025 (2)	−0.036 (2)
C1	0.0371 (16)	0.0371 (16)	0.0486 (17)	−0.0163 (13)	0.0094 (13)	−0.0175 (13)
C2	0.0381 (15)	0.0305 (14)	0.0376 (14)	−0.0175 (12)	0.0127 (11)	−0.0159 (11)
C3	0.0458 (17)	0.0439 (17)	0.0396 (15)	−0.0267 (14)	0.0197 (13)	−0.0184 (13)
C4	0.061 (2)	0.0400 (16)	0.0306 (14)	−0.0269 (15)	0.0192 (13)	−0.0103 (12)
C5	0.0502 (17)	0.0357 (15)	0.0283 (13)	−0.0145 (14)	0.0100 (12)	−0.0080 (11)
C6	0.0346 (14)	0.0301 (14)	0.0298 (13)	−0.0096 (12)	0.0083 (11)	−0.0111 (11)
C7	0.0449 (18)	0.079 (2)	0.0377 (17)	−0.0127 (17)	−0.0014 (13)	−0.0106 (16)
C8	0.0343 (15)	0.0438 (17)	0.0382 (15)	−0.0050 (13)	0.0025 (12)	−0.0126 (13)
C9	0.0297 (15)	0.059 (2)	0.0505 (18)	−0.0125 (15)	0.0058 (13)	−0.0125 (15)
C10	0.0368 (16)	0.0527 (19)	0.0427 (16)	−0.0184 (14)	0.0120 (13)	−0.0122 (14)
C11	0.051 (2)	0.094 (3)	0.0475 (19)	−0.033 (2)	0.0153 (15)	−0.0018 (18)
C12	0.0457 (17)	0.0393 (17)	0.0363 (15)	−0.0180 (14)	0.0087 (12)	−0.0154 (13)
C13	0.0394 (15)	0.0346 (15)	0.0331 (14)	−0.0148 (13)	0.0073 (11)	−0.0106 (12)
C14	0.060 (2)	0.0345 (16)	0.0375 (15)	−0.0177 (15)	0.0046 (13)	−0.0063 (12)
C15	0.073 (2)	0.0425 (18)	0.0271 (14)	−0.0179 (16)	0.0030 (14)	−0.0014 (13)
C16	0.064 (2)	0.0482 (19)	0.0261 (14)	−0.0197 (16)	0.0066 (13)	−0.0113 (13)
C17	0.0342 (14)	0.0359 (15)	0.0292 (13)	−0.0128 (12)	0.0093 (11)	−0.0113 (11)
C18	0.096 (3)	0.065 (2)	0.0479 (19)	−0.021 (2)	0.0194 (18)	−0.0344 (18)
C19	0.0505 (18)	0.0485 (18)	0.0416 (16)	−0.0219 (15)	0.0131 (13)	−0.0250 (14)
C20	0.063 (2)	0.0414 (18)	0.0588 (19)	−0.0210 (16)	0.0165 (16)	−0.0273 (15)
C21	0.0476 (17)	0.0351 (16)	0.0500 (17)	−0.0160 (14)	0.0143 (13)	−0.0133 (13)
C22	0.089 (3)	0.0366 (18)	0.062 (2)	−0.0183 (19)	0.0154 (19)	−0.0035 (16)
C23	0.0583 (19)	0.0384 (17)	0.0365 (16)	−0.0159 (15)	0.0096 (13)	−0.0158 (14)
C24	0.0482 (17)	0.0297 (14)	0.0320 (14)	−0.0077 (13)	0.0086 (12)	−0.0131 (12)
C25	0.0493 (17)	0.0329 (15)	0.0417 (16)	−0.0115 (14)	0.0125 (13)	−0.0179 (13)
C26	0.0576 (19)	0.0339 (16)	0.0355 (15)	−0.0114 (15)	0.0144 (13)	−0.0121 (12)
C27	0.0533 (19)	0.0459 (18)	0.0347 (15)	−0.0061 (15)	0.0073 (13)	−0.0179 (14)
C28	0.0461 (17)	0.0401 (16)	0.0446 (16)	−0.0108 (14)	0.0102 (13)	−0.0223 (14)
C29	0.0500 (17)	0.0363 (16)	0.0364 (15)	−0.0103 (14)	0.0111 (13)	−0.0142 (12)
C30	0.0493 (19)	0.054 (2)	0.0444 (19)	−0.0088 (16)	0.0067 (14)	−0.0078 (16)
C31	0.0389 (17)	0.055 (2)	0.0418 (17)	−0.0087 (15)	0.0049 (13)	−0.0035 (15)
C32	0.0439 (19)	0.063 (2)	0.050 (2)	−0.0131 (17)	0.0055 (14)	−0.0094 (17)
C33	0.0432 (19)	0.082 (3)	0.0407 (18)	−0.0164 (19)	0.0057 (14)	−0.0001 (18)
C34	0.045 (2)	0.067 (3)	0.058 (2)	−0.0190 (19)	0.0007 (16)	0.0136 (19)
C35	0.0429 (19)	0.052 (2)	0.071 (2)	−0.0156 (16)	−0.0008 (16)	−0.0062 (18)
C36	0.0446 (18)	0.059 (2)	0.0497 (18)	−0.0141 (16)	0.0008 (14)	−0.0062 (16)

### Geometric parameters (Å, °)

**Table:**

Mn1—O1	2.1837 (19)	C7—H7A	0.9600
Mn1—O3	2.167 (2)	C7—H7B	0.9600
Mn1—N1	2.207 (2)	C7—H7C	0.9600
Mn1—N3	2.215 (2)	C8—C9	1.365 (4)
Mn1—N4	2.222 (2)	C9—C10	1.397 (4)
Mn1—N6	2.282 (2)	C9—H9	0.9300
N1—C6	1.329 (3)	C10—C11	1.495 (4)
N1—C2	1.343 (3)	C11—H11A	0.9600
N2—C8	1.367 (3)	C11—H11B	0.9600
N2—N3	1.383 (3)	C11—H11C	0.9600
N2—C6	1.413 (3)	C12—C13	1.512 (4)
N3—C10	1.322 (3)	C13—C14	1.379 (4)
N4—C17	1.323 (3)	C14—C15	1.381 (4)
N4—C13	1.338 (3)	C14—H14	0.9300
N5—C19	1.369 (3)	C15—C16	1.375 (4)
N5—N6	1.378 (3)	C15—H15	0.9300
N5—C17	1.414 (3)	C16—C17	1.384 (4)
N6—C21	1.328 (4)	C16—H16	0.9300
N7—O7	1.206 (4)	C18—C19	1.488 (4)
N7—O8	1.209 (4)	C18—H18A	0.9600
N7—C26	1.475 (4)	C18—H18B	0.9600
N8—O9	1.212 (3)	C18—H18C	0.9600
N8—O10	1.228 (3)	C19—C20	1.358 (4)
N8—C28	1.474 (4)	C20—C21	1.386 (4)
N9—O13	1.217 (5)	C20—H20	0.9300
N9—O14	1.217 (5)	C21—C22	1.502 (4)
N9—C33	1.471 (5)	C22—H22A	0.9600
N10—O16	1.213 (5)	C22—H22B	0.9600
N10—O15	1.224 (4)	C22—H22C	0.9600
N10—C35	1.477 (5)	C23—C24	1.503 (4)
O1—C1	1.290 (3)	C24—C25	1.388 (4)
O2—C1	1.215 (3)	C24—C29	1.389 (4)
O3—C12	1.257 (3)	C25—C26	1.372 (4)
O4—C12	1.247 (3)	C25—H25	0.9300
O5—C23	1.192 (3)	C26—C27	1.372 (4)
O6—C23	1.283 (3)	C27—C28	1.386 (4)
O6—H6	0.8200	C27—H27	0.9300
O11—C30	1.197 (4)	C28—C29	1.371 (4)
O12—C30	1.306 (4)	C29—H29	0.9300
O12—H12	0.8200	C30—C31	1.513 (4)
C1—C2	1.515 (4)	C31—C36	1.372 (5)
C2—C3	1.376 (3)	C31—C32	1.382 (4)
C3—C4	1.383 (4)	C32—C33	1.379 (5)
C3—H3	0.9300	C32—H32	0.9300
C4—C5	1.378 (4)	C33—C34	1.373 (5)
C4—H4	0.9300	C34—C35	1.375 (5)
C5—C6	1.384 (3)	C34—H34	0.9300
C5—H5	0.9300	C35—C36	1.386 (4)
C7—C8	1.497 (4)	C36—H36	0.9300
			
O3—Mn1—O1	93.24 (8)	C10—C11—H11C	109.5
O3—Mn1—N1	106.48 (7)	H11A—C11—H11C	109.5
O1—Mn1—N1	72.91 (7)	H11B—C11—H11C	109.5
O3—Mn1—N3	96.33 (8)	O4—C12—O3	126.0 (3)
O1—Mn1—N3	143.17 (7)	O4—C12—C13	117.8 (2)
N1—Mn1—N3	70.27 (8)	O3—C12—C13	116.2 (2)
O3—Mn1—N4	72.54 (7)	N4—C13—C14	121.6 (2)
O1—Mn1—N4	96.62 (7)	N4—C13—C12	113.2 (2)
N1—Mn1—N4	169.48 (8)	C14—C13—C12	125.2 (3)
N3—Mn1—N4	120.18 (8)	C13—C14—C15	118.0 (3)
O3—Mn1—N6	141.46 (7)	C13—C14—H14	121.0
O1—Mn1—N6	97.83 (8)	C15—C14—H14	121.0
N1—Mn1—N6	112.06 (8)	C16—C15—C14	120.6 (3)
N3—Mn1—N6	96.48 (8)	C16—C15—H15	119.7
N4—Mn1—N6	69.58 (8)	C14—C15—H15	119.7
C6—N1—C2	120.0 (2)	C15—C16—C17	117.6 (3)
C6—N1—Mn1	122.45 (17)	C15—C16—H16	121.2
C2—N1—Mn1	117.55 (16)	C17—C16—H16	121.2
C8—N2—N3	110.7 (2)	N4—C17—C16	122.3 (3)
C8—N2—C6	132.6 (2)	N4—C17—N5	113.2 (2)
N3—N2—C6	116.5 (2)	C16—C17—N5	124.5 (2)
C10—N3—N2	105.5 (2)	C19—C18—H18A	109.5
C10—N3—Mn1	134.82 (18)	C19—C18—H18B	109.5
N2—N3—Mn1	117.56 (15)	H18A—C18—H18B	109.5
C17—N4—C13	119.9 (2)	C19—C18—H18C	109.5
C17—N4—Mn1	122.50 (18)	H18A—C18—H18C	109.5
C13—N4—Mn1	116.55 (16)	H18B—C18—H18C	109.5
C19—N5—N6	111.0 (2)	C20—C19—N5	105.6 (2)
C19—N5—C17	131.8 (2)	C20—C19—C18	128.9 (3)
N6—N5—C17	117.1 (2)	N5—C19—C18	125.4 (3)
C21—N6—N5	105.2 (2)	C19—C20—C21	107.7 (3)
C21—N6—Mn1	138.08 (18)	C19—C20—H20	126.1
N5—N6—Mn1	116.41 (16)	C21—C20—H20	126.1
O7—N7—O8	124.3 (3)	N6—C21—C20	110.5 (3)
O7—N7—C26	117.8 (3)	N6—C21—C22	120.5 (3)
O8—N7—C26	117.9 (3)	C20—C21—C22	129.0 (3)
O9—N8—O10	123.9 (3)	C21—C22—H22A	109.5
O9—N8—C28	118.4 (3)	C21—C22—H22B	109.5
O10—N8—C28	117.7 (3)	H22A—C22—H22B	109.5
O13—N9—O14	124.7 (5)	C21—C22—H22C	109.5
O13—N9—C33	118.4 (4)	H22A—C22—H22C	109.5
O14—N9—C33	116.9 (5)	H22B—C22—H22C	109.5
O16—N10—O15	124.5 (5)	O5—C23—O6	126.1 (3)
O16—N10—C35	118.2 (4)	O5—C23—C24	121.3 (3)
O15—N10—C35	117.3 (4)	O6—C23—C24	112.6 (2)
C1—O1—Mn1	120.36 (17)	C25—C24—C29	119.6 (3)
C12—O3—Mn1	120.21 (17)	C25—C24—C23	121.4 (3)
C23—O6—H6	109.5	C29—C24—C23	119.0 (2)
C30—O12—H12	109.5	C26—C25—C24	118.8 (3)
O2—C1—O1	126.0 (3)	C26—C25—H25	120.6
O2—C1—C2	119.3 (3)	C24—C25—H25	120.6
O1—C1—C2	114.7 (2)	C27—C26—C25	123.3 (3)
N1—C2—C3	121.4 (2)	C27—C26—N7	118.1 (3)
N1—C2—C1	114.4 (2)	C25—C26—N7	118.6 (3)
C3—C2—C1	124.1 (2)	C26—C27—C28	116.5 (3)
C2—C3—C4	118.1 (3)	C26—C27—H27	121.7
C2—C3—H3	120.9	C28—C27—H27	121.7
C4—C3—H3	120.9	C29—C28—C27	122.4 (3)
C5—C4—C3	120.8 (2)	C29—C28—N8	119.0 (3)
C5—C4—H4	119.6	C27—C28—N8	118.6 (3)
C3—C4—H4	119.6	C28—C29—C24	119.4 (3)
C4—C5—C6	117.5 (3)	C28—C29—H29	120.3
C4—C5—H5	121.2	C24—C29—H29	120.3
C6—C5—H5	121.2	O11—C30—O12	126.2 (3)
N1—C6—C5	122.1 (2)	O11—C30—C31	120.9 (3)
N1—C6—N2	112.6 (2)	O12—C30—C31	112.8 (3)
C5—C6—N2	125.3 (2)	C36—C31—C32	120.7 (3)
C8—C7—H7A	109.5	C36—C31—C30	118.2 (3)
C8—C7—H7B	109.5	C32—C31—C30	121.0 (3)
H7A—C7—H7B	109.5	C33—C32—C31	118.6 (4)
C8—C7—H7C	109.5	C33—C32—H32	120.7
H7A—C7—H7C	109.5	C31—C32—H32	120.7
H7B—C7—H7C	109.5	C34—C33—C32	122.6 (3)
C9—C8—N2	106.2 (2)	C34—C33—N9	119.0 (4)
C9—C8—C7	128.2 (3)	C32—C33—N9	118.4 (4)
N2—C8—C7	125.6 (3)	C33—C34—C35	117.0 (3)
C8—C9—C10	106.9 (3)	C33—C34—H34	121.5
C8—C9—H9	126.5	C35—C34—H34	121.5
C10—C9—H9	126.5	C34—C35—C36	122.4 (4)
N3—C10—C9	110.6 (2)	C34—C35—N10	118.9 (3)
N3—C10—C11	120.0 (3)	C36—C35—N10	118.6 (4)
C9—C10—C11	129.4 (3)	C31—C36—C35	118.6 (3)
C10—C11—H11A	109.5	C31—C36—H36	120.7
C10—C11—H11B	109.5	C35—C36—H36	120.7
H11A—C11—H11B	109.5		
			
O3—Mn1—N1—C6	−88.9 (2)	Mn1—N3—C10—C9	162.0 (2)
O1—Mn1—N1—C6	−177.2 (2)	N2—N3—C10—C11	179.3 (3)
N3—Mn1—N1—C6	2.06 (19)	Mn1—N3—C10—C11	−18.2 (5)
N4—Mn1—N1—C6	−171.9 (4)	C8—C9—C10—N3	0.1 (4)
N6—Mn1—N1—C6	91.1 (2)	C8—C9—C10—C11	−179.7 (3)
O3—Mn1—N1—C2	90.78 (19)	Mn1—O3—C12—O4	174.0 (2)
O1—Mn1—N1—C2	2.46 (17)	Mn1—O3—C12—C13	−6.3 (3)
N3—Mn1—N1—C2	−178.3 (2)	C17—N4—C13—C14	0.2 (4)
N4—Mn1—N1—C2	7.7 (5)	Mn1—N4—C13—C14	−168.2 (2)
N6—Mn1—N1—C2	−89.21 (19)	C17—N4—C13—C12	178.8 (2)
C8—N2—N3—C10	0.7 (3)	Mn1—N4—C13—C12	10.4 (3)
C6—N2—N3—C10	175.6 (2)	O4—C12—C13—N4	176.7 (3)
C8—N2—N3—Mn1	−165.39 (18)	O3—C12—C13—N4	−3.0 (4)
C6—N2—N3—Mn1	9.6 (3)	O4—C12—C13—C14	−4.8 (4)
O3—Mn1—N3—C10	−61.7 (3)	O3—C12—C13—C14	175.5 (3)
O1—Mn1—N3—C10	−165.8 (2)	N4—C13—C14—C15	1.3 (4)
N1—Mn1—N3—C10	−167.0 (3)	C12—C13—C14—C15	−177.1 (3)
N4—Mn1—N3—C10	11.8 (3)	C13—C14—C15—C16	−0.7 (5)
N6—Mn1—N3—C10	81.9 (3)	C14—C15—C16—C17	−1.3 (5)
O3—Mn1—N3—N2	99.20 (18)	C13—N4—C17—C16	−2.4 (4)
O1—Mn1—N3—N2	−4.9 (3)	Mn1—N4—C17—C16	165.3 (2)
N1—Mn1—N3—N2	−6.07 (17)	C13—N4—C17—N5	179.1 (2)
N4—Mn1—N3—N2	172.66 (16)	Mn1—N4—C17—N5	−13.2 (3)
N6—Mn1—N3—N2	−117.22 (18)	C15—C16—C17—N4	2.9 (4)
O3—Mn1—N4—C17	−178.2 (2)	C15—C16—C17—N5	−178.8 (3)
O1—Mn1—N4—C17	−86.9 (2)	C19—N5—C17—N4	−166.3 (3)
N1—Mn1—N4—C17	−91.9 (5)	N6—N5—C17—N4	9.8 (3)
N3—Mn1—N4—C17	94.6 (2)	C19—N5—C17—C16	15.2 (5)
N6—Mn1—N4—C17	9.06 (19)	N6—N5—C17—C16	−168.7 (3)
O3—Mn1—N4—C13	−10.18 (18)	N6—N5—C19—C20	0.5 (3)
O1—Mn1—N4—C13	81.15 (19)	C17—N5—C19—C20	176.8 (3)
N1—Mn1—N4—C13	76.1 (5)	N6—N5—C19—C18	−175.9 (3)
N3—Mn1—N4—C13	−97.4 (2)	C17—N5—C19—C18	0.4 (5)
N6—Mn1—N4—C13	177.1 (2)	N5—C19—C20—C21	−0.8 (4)
C19—N5—N6—C21	−0.1 (3)	C18—C19—C20—C21	175.6 (3)
C17—N5—N6—C21	−177.0 (2)	N5—N6—C21—C20	−0.4 (3)
C19—N5—N6—Mn1	174.34 (18)	Mn1—N6—C21—C20	−172.9 (2)
C17—N5—N6—Mn1	−2.5 (3)	N5—N6—C21—C22	178.1 (3)
O3—Mn1—N6—C21	157.9 (3)	Mn1—N6—C21—C22	5.5 (5)
O1—Mn1—N6—C21	−96.7 (3)	C19—C20—C21—N6	0.7 (4)
N1—Mn1—N6—C21	−22.1 (3)	C19—C20—C21—C22	−177.6 (3)
N3—Mn1—N6—C21	49.2 (3)	O5—C23—C24—C25	159.6 (3)
N4—Mn1—N6—C21	169.1 (3)	O6—C23—C24—C25	−18.8 (4)
O3—Mn1—N6—N5	−14.0 (2)	O5—C23—C24—C29	−20.2 (4)
O1—Mn1—N6—N5	91.29 (17)	O6—C23—C24—C29	161.5 (3)
N1—Mn1—N6—N5	165.96 (16)	C29—C24—C25—C26	0.0 (4)
N3—Mn1—N6—N5	−122.74 (17)	C23—C24—C25—C26	−179.7 (3)
N4—Mn1—N6—N5	−2.90 (16)	C24—C25—C26—C27	−1.3 (4)
O3—Mn1—O1—C1	−108.8 (2)	C24—C25—C26—N7	179.2 (2)
N1—Mn1—O1—C1	−2.53 (19)	O7—N7—C26—C27	170.2 (3)
N3—Mn1—O1—C1	−3.7 (3)	O8—N7—C26—C27	−9.5 (4)
N4—Mn1—O1—C1	178.4 (2)	O7—N7—C26—C25	−10.2 (4)
N6—Mn1—O1—C1	108.2 (2)	O8—N7—C26—C25	170.1 (3)
O1—Mn1—O3—C12	−87.1 (2)	C25—C26—C27—C28	1.7 (4)
N1—Mn1—O3—C12	−160.2 (2)	N7—C26—C27—C28	−178.7 (3)
N3—Mn1—O3—C12	128.5 (2)	C26—C27—C28—C29	−1.0 (4)
N4—Mn1—O3—C12	8.8 (2)	C26—C27—C28—N8	−179.9 (3)
N6—Mn1—O3—C12	19.8 (3)	O9—N8—C28—C29	−173.6 (3)
Mn1—O1—C1—O2	−179.6 (2)	O10—N8—C28—C29	6.1 (4)
Mn1—O1—C1—C2	2.2 (3)	O9—N8—C28—C27	5.3 (4)
C6—N1—C2—C3	−0.3 (4)	O10—N8—C28—C27	−175.0 (3)
Mn1—N1—C2—C3	−179.96 (19)	C27—C28—C29—C24	−0.1 (4)
C6—N1—C2—C1	177.4 (2)	N8—C28—C29—C24	178.8 (3)
Mn1—N1—C2—C1	−2.2 (3)	C25—C24—C29—C28	0.6 (4)
O2—C1—C2—N1	−178.3 (3)	C23—C24—C29—C28	−179.7 (2)
O1—C1—C2—N1	0.1 (3)	O11—C30—C31—C36	−0.5 (5)
O2—C1—C2—C3	−0.6 (4)	O12—C30—C31—C36	−178.5 (3)
O1—C1—C2—C3	177.7 (2)	O11—C30—C31—C32	178.0 (3)
N1—C2—C3—C4	−1.1 (4)	O12—C30—C31—C32	0.0 (4)
C1—C2—C3—C4	−178.6 (3)	C36—C31—C32—C33	−0.1 (5)
C2—C3—C4—C5	1.3 (4)	C30—C31—C32—C33	−178.5 (3)
C3—C4—C5—C6	−0.2 (4)	C31—C32—C33—C34	1.4 (5)
C2—N1—C6—C5	1.5 (4)	C31—C32—C33—N9	−179.0 (3)
Mn1—N1—C6—C5	−178.84 (19)	O13—N9—C33—C34	−170.1 (4)
C2—N1—C6—N2	−177.5 (2)	O14—N9—C33—C34	9.1 (5)
Mn1—N1—C6—N2	2.1 (3)	O13—N9—C33—C32	10.2 (6)
C4—C5—C6—N1	−1.3 (4)	O14—N9—C33—C32	−170.6 (4)
C4—C5—C6—N2	177.7 (3)	C32—C33—C34—C35	−2.1 (5)
C8—N2—C6—N1	166.1 (3)	N9—C33—C34—C35	178.2 (3)
N3—N2—C6—N1	−7.5 (3)	C33—C34—C35—C36	1.6 (5)
C8—N2—C6—C5	−12.9 (5)	C33—C34—C35—N10	179.8 (3)
N3—N2—C6—C5	173.5 (2)	O16—N10—C35—C34	171.9 (4)
N3—N2—C8—C9	−0.6 (3)	O15—N10—C35—C34	−6.1 (5)
C6—N2—C8—C9	−174.5 (3)	O16—N10—C35—C36	−9.9 (5)
N3—N2—C8—C7	178.5 (3)	O15—N10—C35—C36	172.2 (3)
C6—N2—C8—C7	4.7 (5)	C32—C31—C36—C35	−0.4 (5)
N2—C8—C9—C10	0.3 (3)	C30—C31—C36—C35	178.1 (3)
C7—C8—C9—C10	−178.8 (3)	C34—C35—C36—C31	−0.4 (5)
N2—N3—C10—C9	−0.4 (3)	N10—C35—C36—C31	−178.6 (3)

### Hydrogen-bond geometry (Å, °)

**Table:**

*D*—H···*A*	*D*—H	H···*A*	*D*···*A*	*D*—H···*A*
O6—H6···O4	0.82	1.76	2.575 (3)	171
O12—H12···O1	0.82	1.76	2.552 (3)	162
